# Nurses’ Perspectives on Unmet Social, Psychological, and Spiritual Needs of Palliative Patients in Croatia: A Cross-Sectional Study

**DOI:** 10.3390/nursrep16010029

**Published:** 2026-01-16

**Authors:** Ana Ćurković, Matea Dolić, Linda Lušić Kalcina

**Affiliations:** 1 Faculty of Health Sciences, University of Split, Ruđera Boškovića 35, 21000 Split, Croatia; acurkovic@ozs.unist.hr; 2 School of Medicine, University of Split, Šoltanska 2A, 21000 Split, Croatia

**Keywords:** palliative care, social needs, psychological needs, spiritual needs, Croatia, nurse perceptions

## Abstract

**Background:** Palliative care addresses not only physical symptoms but also the social, psychological, and spiritual needs of patients. Nurses play a key role in identifying and responding to these needs, yet their perceptions and preparedness may vary. **Objectives:** This study aimed to explore nurses’ perspectives on the psychological, social, and spiritual needs of palliative patients, assess how well these needs are being met, and examine the influence of nurses’ self-assessed education levels on their evaluations. **Methods:** A cross-sectional survey was conducted among 237 registered nurses with palliative care experience in Split-Dalmatia County, Croatia. Two validated questionnaires were used to assess the perceived importance of 53 patient needs and the extent to which these needs were satisfied. **Results:** Findings revealed significant discrepancies between the perceived importance and satisfaction of nearly all psychological, social, and spiritual needs (*p* < 0.001), particularly regarding fear of death, suffering, and future uncertainty. Only 38.4% of nurses considered themselves adequately trained in palliative care, though most had some educational exposure to it. No statistical differences were found in need assessment based on nurses’ self-rated education. Most nurses reported emotional exhaustion (72.6%) and supported interdisciplinary care (95.8%), while 90.3% noted that responsibility for care often falls on families. **Conclusions:** Nurses recognize critical unmet needs in palliative patients and feel insufficiently prepared to address them. These findings underscore the need to improve palliative care education, provide emotional support for nurses, and implement systemic healthcare reforms to ensure comprehensive, dignified care.

## 1. Introduction

Palliative medicine is a relatively new branch of medicine focused on ensuring optimal quality of life for patients with incurable diseases. It encompasses comprehensive, multidisciplinary care aimed at alleviating suffering and improving the quality of life for patients whose disease no longer responds to curative treatment [1,2]. Such care extends beyond medical treatment and symptom control, including pain, shortness of breath, and nausea. It also involves psychological support to help patients and their families cope with emotional distress, as well as social support for practical concerns such as financial or legal issues and access to community services. In addition, spiritual care supports patients in finding meaning and peace, often through contact with spiritual advisors or religious practices [3,4]. This holistic approach ensures that patients are treated as whole persons, emphasizing the importance of autonomy and preserving the dignity of patients until the natural end of life. To this end, interdisciplinary teams, including doctors, nurses, psychologists, social workers, volunteers, and other specialists, work together to plan and implement interventions tailored to the individual needs of patients [5,6].

Palliative care implies the art and science of assisting people at the end of their life, helping them meet basic human needs, achieve the highest possible level of independence, and attain optimal quality of life. Palliative care can be provided in various settings: hospitals, hospices, long-term care facilities, or even at home. The World Health Organization defines palliative care as active and comprehensive care for patients whose disease no longer responds to treatment [7,8,9]. Palliative care focuses on relieving pain and other distressing symptoms while addressing the psychological, social, and spiritual challenges experienced by patients and their families, with the overarching aim of optimizing quality of life [8,10,11]. Through support systems that address fundamental aspects of daily living, palliative care helps patients live actively until the moment of death [7]. According to one of the most influential theorists of modern nursing, Virginia Henderson, basic human needs form a framework that allows for comprehensive care. When a patient cannot independently meet these needs, such as breathing, nutrition, elimination, movement, personal hygiene, sleep and rest, dressing and changing clothes, maintaining body temperature, bodily cleanliness, avoiding environmental dangers, communication, spiritual care, work and a sense of achievement, play or recreation, learning, and personal development, the nurse assumes the role of assistant, ensuring that the patient retains dignity and quality of life [12,13,14].

Patients remain spiritual beings even when disoriented, confused, emotionally ill, irrational, or intellectually impaired. Spiritual needs include seeking meaning, hope, connection, forgiveness, and acceptance, and do not necessarily relate to religiosity. It is necessary to distinguish between spirituality and religiosity in the context of value systems. Spirituality is defined as a person’s ability to experience meaning in themselves and others, including, for example, music, literature, nature, or anything considered a greater force than their self-perception [15]. Every patient with a severe, incurable illness experiences spiritual pain and has spiritual needs [16]. These needs include seeking the meaning of life and experiences associated with the approach of death, as well as being perceived as a whole person. They also involve addressing existential questions and making appropriate moral decisions, achieving reconciliation and unity with others, expressing final farewells in peace, respecting their way of life, and realizing their spiritual potential. In moments of suffering, the patient wants to be loved and accepted and faces questions about the meaning of life and the nature of their suffering. Every request, need, and call expresses a need for love and closeness [17,18,19,20,21]. Through the development of trust, the patient can accept reality, finding meaning in what they are experiencing. Nurses create an environment where patients feel free to express their feelings, fears, anxieties, and questions [14,22]. The purpose is to enable individuals to be completely at ease, allowing them to enter a phase of dignified death. Every patient’s journey is unique. Palliative care teams work closely with patients and their families to understand their values, preferences, and goals. This personalized approach helps ensure that treatment and intervention decisions align with what matters most to the individual, whether staying at home, engaging in social activities, or achieving a peaceful, dignified end-of-life experience [7,10,23].

Harrad et al. (2019) found that nurses do not feel adequately prepared to provide spiritual care, considering it part of patient privacy [24]. They also lack sufficient knowledge about spirituality and religious beliefs, viewing these needs as the exclusive domain of pastoral representatives of certain religious communities. Healthcare professionals frequently encounter ethical and legal dilemmas in their practice, which may lead to an increased sense of moral distress. This is particularly evident in situations involving the care of patients with severe conditions, such as brain death [25,26,27]. Recent studies indicate that such circumstances often give rise to conflicts between professional obligations, legal regulations, and personal conscience. Furthermore, evidence suggests that young nurses often possess insufficient knowledge, especially regarding legal frameworks, and that without adequate support in medical and ethical–legal aspects of care, they are more likely to face dilemmas that may result in moral distress [28,29]. Nurses’ perceptions of their education in palliative care significantly influence the quality of care provided to palliative patients. When nurses feel unprepared, the gap between patients’ needs and provided care widens. Conversely, targeted and comprehensive educational interventions have been shown to improve nurses’ knowledge and attitudes, leading to better patient outcomes. Systematic assessment of educational needs, using tools that measure both knowledge and attitudes, is essential for developing training programs that ultimately enhance the palliative care experience for patients and their families [30,31]. Several studies have evaluated the impact of educational interventions in palliative care on nurses’ attitudes and competencies. For example, a pre- and post-study of a dedicated palliative care course for nursing students showed that participants improved not only their knowledge but also developed more positive attitudes towards caring for dying patients [32,33,34,35]. However, nurses’ roles also include understanding religion and the religious needs important to patients and their family members to ensure their availability and adequate fulfillment [36,37,38].

The aim of this study was to examine nurses’ perceptions of the psychological, social, and spiritual needs of palliative patients and the extent to which these needs are met.

The specific objectives were: (1) to assess nurses’ evaluations of the importance and satisfaction of these needs, and (2) to determine whether these evaluations differ according to nurses’ self-assessed level of palliative care education.

## 2. Materials and Methods

### 2.1. Study Design

This cross-sectional study was conducted as a part of the institutional project “Assessment of Psychological and Spiritual Issues in Palliative Patients in Split-Dalmatia County” (SOZS-IP-2022-1). The study targeted a convenience sample of nurses who provide care to palliative patients. The questionnaires were administered online via Google Forms (Google LLC, Mountain View, CA, USA), and the link was sent to nurses employed at the University Hospital of Split and the Split Health Centre, which also includes hospice and nursing homes.

### 2.2. Sample

The sample was convenient and consisted of 237 female nurses who provide or have been providing care to palliative patients at any level of their work experience. The inclusion criteria were being a nurse and having experience in providing care to palliative patients. Exclusion criteria were not being a registered nurse and not having experience in palliative care. Experience in palliative care was defined according to Janice Monti’s recommendations [39], which describe palliative care nurses as those who offer comfort to terminally ill patients, curative care to critically ill patients, and support to patients’ families. They serve patients of all ages, including those with life-threatening injuries or disabilities that need continuous care, as well as illnesses like dementia, kidney failure, heart disease, and cancer. In Croatia, there is no specialization for nurses in the field of palliative care. The nurses in our sample all had a general profile and had experience working with palliative patients in their respective job roles. When answering the questions, the nurses considered palliative patients with various diagnoses who retained their cognitive abilities.

### 2.3. Questionnaires

In this study, we used two questionnaires: Psychological, Social, and Spiritual Problems of Palliative Patients’ Questionnaire [40] and the Effectiveness in Coping with the Psychological, Social, and Spiritual Challenges of Palliative Care Patients [40]. Those instruments were previously developed and validated by our team through a four-phase process including content validation by a multidisciplinary expert group, linguistic validation via double translation, and construct validation through exploratory factor analysis (EFA). The EFA identified a four-factor structure for the first questionnaire (64.56% explained variance) and a three-factor structure for the second (69.97%). The adequacy of the data for factor analysis was confirmed by high Kaiser–Meyer–Olkin (KMO) values (0.952 and 0.963) and statistically significant Bartlett’s tests of sphericity (*p* < 0.001). Both instruments demonstrated excellent internal consistency, with Cronbach’s alpha values of 0.98 and 0.985, respectively, indicating a high degree of reliability. These results confirm the strong psychometric properties of both questionnaires [40]. Although exploratory factor analysis confirmed a multi-factor structure of both instruments, total or factor scores were not computed because each of the 53 items represents a distinct palliative care need. The aim of this study was to identify differences at the level of individual needs rather than to aggregate them into summary scores.

Those two questionnaires assess nurses’ perspectives on the psychological, social, and spiritual needs of palliative care patients. Both questionnaires consisted of 53 items. The Psychological, Social, and Spiritual Problems of Palliative Patients’ Questionnaire [40] evaluates the psychological, social, and spiritual needs of palliative patients as assessed from the perspective of nurses who provide palliative care. The Effectiveness in Coping with Psychological, Social, and Spiritual Challenges of Palliative Care Patients [40] assesses how effectively these challenges are being addressed from the perspective of nurses who provide palliative care. Nurses evaluated the needs and the effectiveness of addressing them with a 5-point Likert scale. In the first questionnaire, responses ranged from 1 (“Not a problem at all”) to 5 (“It is the greatest difficulty/unmet need for most palliative care patients”). In the second questionnaire, responses ranged from 1 (“Not satisfied at all”) to 5 (“Completely satisfied”). These two 53-item questionnaires were used to examine differences between perceived importance and satisfaction with palliative patients’ psychological, social, and spiritual needs. Additionally, nurses were asked a few demographic questions (gender, number of years working with palliative patients, and institution/department of employment), as well as 11 yes/no questions assessing their self-perceived competence in palliative care, attitudes toward education in this area, and perceptions of work-related challenges. These questions were used to evaluate whether the nurses’ perception of palliative patient needs and the extent to which these needs are met differed according to their self-assessed level of palliative care training. Questions related to emotional exhaustion and work-related burden were included for descriptive and contextual purposes only and were not intended to assess psychological disorders.

### 2.4. Statistical Analysis

Descriptive statistics included frequencies, percentages, and median with interquartile range. The Kolmogorov–Smirnov test was performed to assess the normality of the data distribution. Since the data were not normally distributed, non-parametric statistical tests were applied throughout the analysis.

The Mann–Whitney U test and Chi-Square test were used to compare responses about nurses’ education and experience in palliative care and their perceptions and attitudes about palliative care between two groups: nurses who considered themselves adequately trained vs. those who did not. These results are presented in Table 1.

The Wilcoxon signed-rank test was used to compare paired data from the same respondents: each nurse’s ratings of the importance of a need, and their ratings of the satisfaction of that need. These results are presented in Table 2, Table 3 and Table 4. To compare the assessment of palliative patients’ needs between nurses who considered themselves adequately trained in palliative care and those who did not, a Mann–Whitney U test was again applied. These results are presented in the Supplementary Material (Tables S1–S3).

The significance level was set at *p* < 0.05. Statistical analyses were conducted using IBM SPSS Statistics version 29.0 Premium Campus Edition (IBM Corp., Armonk, NY, USA).

### 2.5. Ethics

The study was conducted following the Declaration of Helsinki and approved by the ethics committees of the University Hospital of Split (Class: 500-03/22-01/167 No: 2181-147/01/06/M.5.-22-02. September 2022), the Split Health Centre Split (Class: 053-01/22-01l/021 No: 2181-149-18-22-00. September 2022), and the University Department of Health Studies (Class: 029-03/22-08/01 No: 2181-228-103/1-22-32. October 2022). Participation was voluntary and anonymous, with the possibility of refusing to participate or withdrawing at any time after consenting. All nurses who participated were informed about who was conducting the research, as well as the purpose and objectives of the study. Since the research was conducted online, pressing the “Submit” button was considered consent to participate in the research. Instructions for completing the questionnaires were provided to ensure the accuracy of the data.

## 3. Results

### 3.1. Experiences and Perceptions of Nurses Working with Palliative Care Patients

The current study involved 237 female nurses with a median of 7 years of work experience in palliative care (interquartile range = 14 years). For 65.8% of them, working with palliative care patients is part of their daily duties, while only 38.4% consider themselves adequately trained for palliative care. Among the total number of nurses, 68.4% reported receiving education in palliative care during their formal education through attending and completing courses in this field, while 38.4% continued their education in palliative care through lifelong learning programs. In line with these findings, the majority of respondents believe that more attention should be given to the field of palliative care in formal education (84.8%) and that conferences and workshops in this field should be organized more frequently (75.1%). Additionally, 72.6% of nurses find working with palliative care patients to be emotionally exhausting, and 92.8% believe that psychological support should be provided to professionals working in palliative care. Nearly all respondents (95.8%) support an interdisciplinary approach to palliative care, which includes doctors, nurses, psychologists, priests, social workers, and other professionals. However, a concerning 90.3% of respondents indicate that the responsibility for addressing the needs of palliative care patients primarily falls to their families. When comparing nurses who perceived themselves as adequately trained versus those who did not, several statistically significant differences emerged. Nurses who consider themselves adequately trained in palliative care have more years of work experience in the field, and working with palliative patients is more frequently part of their daily responsibilities compared to those who do not consider themselves adequately trained (*p* < 0.001). Additionally, nurses who perceive themselves as adequately trained have more often participated in lifelong learning programs related to palliative care than those who do not feel sufficiently trained (*p* < 0.001). This group also more often believes that working with palliative patients is emotionally draining and that they sometimes need support (*p* < 0.001), but they are also more likely to agree that palliative care teams available within the healthcare system are sufficient to provide adequate care for palliative patients (*p* = 0.006) (Table 1).

### 3.2. Comparison of Perceived Importance and Satisfaction of Needs

We calculated medians and interquartile range for nurses’ assessment of all the 53 palliative patients’ needs importance and satisfaction level from our questionnaires and made a comparison. Of the 53 palliative care needs assessed, only one demonstrated no significant discrepancy between nurses’ evaluations of its importance and the extent to which it was fulfilled. This need falls within the spiritual category and relates to receiving the sacrament of the anointing of the sick without linking it to last rites or impending death. For all the other palliative patients’ spiritual (Table 4), psychological (Table 3) and social needs (Table 2) our results showed a statistically significant difference between the nurses’ assessment of palliative patients’ needs and the nurses’ assessment of the degree to which these needs are met (*p* < 0.001 for most needs, *p* = 0.006 for one need).

The median of the assessed importance of needs is mostly 4.0 or 3.0, while the median of the assessed level of need satisfaction is 3.0 for all needs. The negative Z value of the Wilcoxon Signed Ranks Test indicates that satisfaction ratings are significantly lower than importance ratings. This means there is a significant gap between nurses’ assessment of the importance of palliative patients’ needs and their assessment of the degree to which these needs are satisfied. According to the results, psychological needs are those for which nurses perceive the greatest discrepancy between their importance to palliative patients and the extent to which these needs are being met. The smallest difference, although still statistically significant, was found in the assessment of the importance and satisfaction level of spiritual needs. Among the psychological needs of palliative patients, the needs for which results show the greatest difference between the assessment of importance and the assessment of satisfaction are needs to reduce fears of death (Z = −9.474), physical suffering (Z = −9.363), unpredictability of the future (Z = −9.257), disease progression (Z = −9.219), and loneliness (Z = −9.208) (Table 2).

Among the social needs of palliative patients, the greatest differences between the assessment of importance and satisfaction were recorded for need to ensure the safety of children, in terms of having someone to take care of them (Z = −9.435), need for employment or continuing education (for patients who are not in a terminal phase and do not have dementia) (Z = −9.264), need for relaxation (Z = −8.370), need to accept dependence on others (Z = −8.421), and need to overcome loneliness (Z = −8.098). Among the social needs, the only one with a smaller difference between the assessed importance and satisfaction was the need for more practical help from a partner or family, but even in this case, a statistically significant difference remains (Z = −2.776, *p* = 0.006) (Table 3).

In the assessment of the importance of spiritual needs and the level of their satisfaction, the differences were the smallest, but still statistically significant for all spiritual needs except the need to receive the sacrament of anointing of the sick without associating it with last rites or imminent death. The spiritual needs with the greatest differences between nurses’ assessment of importance and satisfaction levels were need for acceptance of the illness (Z = −8.164), the impossibility of reconciling with certain people who have passed through their life (Z = −7.740), the wrong life choices that cannot be corrected (Z = −7.415), and the lost time or opportunities in life (Z = −7.306) (Table 4).

### 3.3. Differences Based on Nurses’ Self-Assessed Education

In the comparison of the assessment of palliative patients’ needs and their satisfaction levels by nurses who consider themselves adequately trained in palliative care and those who do not, no statistically significant difference was found for most needs. When comparing the assessment of importance level between those two groups, the Mann–Whitney U test showed a statistically significant difference only for one psychological need: the need to reduce the fear of physical suffering (Z = −2.120, *p* = 0.034). The spiritual need for receiving the sacrament of anointing of the sick without associating it with last rites or imminent death showed a borderline statistical significance in the importance assessment between these two groups of nurses (Z = −1.953, *p* = 0.051). For both needs, nurses who do not feel adequately trained in the field of palliative care rate the need as more important compared to those who consider themselves sufficiently trained (mdn = 4.0 vs. mdn = 3.0). For the assessment of the level of satisfaction of palliative patients’ needs by these two groups of nurses, a statistically significant difference was found only for two social needs; the need for employment or continuation of education for patients who are not in terminal stage and do not have dementia (Z = −2.251, *p* = 0.024), where nurses who feel adequately trained in the field of palliative care assess a higher level of satisfaction compared to those who do not feel adequately trained (mdn = 3.0 vs. mdn = 2.0), and the need for significant others not to dramatize the situation (Z = −1.991, *p* = 0.046, mdn = 3.0 for both groups). However, for most needs, no statistically significant differences were found between these two groups of nurses, suggesting that the perception of one’s own education in palliative care has a limited impact on the assessment of the importance and satisfaction of palliative patients’ needs (Tables S1–S3; Supplementary Material).

## 4. Discussion

Although palliative care education exists in Croatia, our findings indicate that nurses still perceive significant gaps in both formal and lifelong training, which aligns with previous studies showing inadequate nurse preparation in this field [41,42,43,44]. The lack of education and competence among nurses affects the quality of care provided to palliative patients [45], which is why it is extremely important to address this issue by improving educational and practical training programs. Through these programs, nurses should acquire both theoretical and practical knowledge and gain the competencies and confidence needed to work in palliative care [45]. Research has shown that educational interventions are an effective tool for improving knowledge, skills, and confidence in the provision of palliative care by nurses [46,47,48,49], so lifelong education should be considered an important tool for enhancing the knowledge and skills of nurses. Furthermore, the results suggest that the lack of clear regulatory frameworks and specific education affects not only the level of knowledge but also the professional autonomy of healthcare workers, frequently giving rise to ethical dilemmas in daily practice. In this context, improving programs in the domains of ethics, law, and spirituality becomes a necessity. Integrating such content into the education system would enable the strengthening of professional competencies and ensure more effective synchronization within the interdisciplinary palliative care team, thereby providing comprehensive support for patients at the end of life [25,28,29].

Most respondents found working with palliative patients emotionally exhausting, and nearly all agreed that psychological support should be available for professionals in this field. Emotional exhaustion is a key factor and trigger of burnout [50,51], and it is particularly common among palliative care workers [52,53]. Burnout can impair nurses’ performance and lower the quality of care provided to patients [51,54,55]. Education and training are crucial for helping nurses recognize emotional strain and early signs of burnout [56], while addressing burnout not only supports nurses’ well-being but also improves patient care outcomes [55].

Nurses in this study recognize the importance of teamwork in palliative care, as nearly all respondents support the establishment of an interdisciplinary team that includes doctors, nurses, clergy, psychologists, and social workers. In line with the European Association for Palliative Care, the Croatian Chamber of Nurses has aligned its palliative care nursing competencies, highlighting the importance of nurses in delivering specialist care, attending to patients’ psychological and spiritual needs, and working in interdisciplinary teams [44,57]. Interdisciplinary teams in palliative care are important for covering various aspects of patients’ needs and for providing proper support for patients and their families [58,59]. Palliative care should adopt a holistic approach by addressing physical, psychological, social, emotional, and spiritual sources of suffering [60]. Palliative patients have specific and multidimensional needs, and that is why this interdisciplinary approach is so important [61,62,63,64]. Besides symptom management, palliative patients need social support, communication, a sense of belonging, and a meaningful life, support to cope with their feelings about dying and death [63], as well as support in dealing with anxiety, depression, grief, and anger [65]. Not addressing these needs can lead to a negative consequence for palliative patients, like increased fear, panic attacks, and anxiety [66], and when those needs are adequately satisfied, patients easily accept their condition and deal with the end of life easily [67]. Therefore, the palliative care team must be thoughtfully assembled and adequately trained to provide the best possible support to the patient.

Unfortunately, nearly all the surveyed nurses confirmed that the care of palliative patients is mostly left to family members. In Croatia, the palliative care system is still in development and does not yet meet the needs of Croatian society. Such an undeveloped palliative care system undermines the principle of equity, as not all citizens have equal access to care [68]. It is estimated that between 26,000 and 46,000 patients in Croatia require some form of palliative care annually, while between 349 and 429 patients require placement in specialized palliative care facilities. Currently, only two hospices are operating in Croatia, with the remaining palliative care beds located within hospitals. Although 363 palliative care beds are available nationwide [69], this capacity is insufficient to meet population needs, indicating a clear need for expanded palliative care infrastructure, including additional dedicated beds and hospice services. Many patients in need of some form of palliative care are placed in homes for the elderly and disabled or remain at their home where they are being cared for by family members. In this way, homes for the elderly and family members take on the role of providing palliative care, although they are often neither trained nor qualified for such care [68]. Family members taking care of palliative patients often feel unprepared for the role of a caregiver and put their own needs aside [70], which highlights the need for better support for family caregivers. Caring for palliative patients at home by family members can be positive for patients, but with the proper support from a palliative care team [71] and education [72]. These findings indicate that special attention should be given to programs focused on educating and preparing family members to care for palliative patients, as well as strengthening close collaboration with the palliative care team. This approach is essential to ensure that the patient receives the best possible care and that their needs are met even within their own home.

A comparison of the assessment and satisfaction of palliative patients’ needs reveals that nurses believe these needs are not being adequately met. Nurses rated the social, psychological, and spiritual needs of palliative patients as more important than how well these needs are currently being met. Some other global studies also show that those needs are often inadequately addressed [73,74,75,76]. Despite global progress in palliative care, significant obstacles remain, including unequal access, limited resources and education, and diverse cultural perspectives on end-of-life care [77]. Additionally, the lack of proper training, insufficient compensation, and healthcare professionals’ discomfort with death are highlighted as key barriers to providing high-quality palliative care [78]. Given that unmet palliative care needs are linked to poorer quality of life and increased use of healthcare services, including more frequent emergency visits, hospitalizations, higher mortality rates [79], and poorer quality of life [80], these findings underscore the importance of addressing gaps in care to better meet the comprehensive needs of palliative patients.

Among the three domains assessed, psychological needs emerged as the most unmet, which is consistent with earlier studies that also identified the most unmet needs of palliative patients in the psychological domain [81,82,83,84]. Those studies show that those needs are particularly related to patients’ fears and concerns, which are centered on uncertainty and fear of suffering [84], such as fear of the uncertain future and the progression of their disease [81,82]. The least well-met social need of palliative patients, according to our nurses, is the need to ensure children’s safety, specifically to secure appropriate care for them. This is to be expected for patients with children, since children are the primary issue for parents to worry about when they receive a negative prognosis. Palliative patients with dependent children have a great need to feel reassured that their children will be taken care of after their death, according to other studies [85,86]. The second social need that nurses rate as the least adequately met is the need to retain employment or continue education among patients who are not in the terminal phase and do not have dementia. This is entirely expected, as palliative patients often require continuous care, and managing chronic illness can be all-consuming [87], making it difficult to maintain regular employment [88]. According to nurses’ assessments, palliative patients struggle with accepting dependence on others, which aligns with other studies showing that palliative patients have a strong desire to remain physically independent [89] and often find it difficult to come to terms with relying on others [90]. When they become dependent, patients may begin to feel a loss of identity and autonomy [91,92], which further contributes to emotional distress and more psychological needs to be met. Nurses also reported that palliative patients have difficulty accepting their illness, and it is particularly important to address this need, as patients who struggle to accept their prognosis often experience higher levels of depression, anxiety, hopelessness, and suffering [93]. A qualitative study by Asghari and Arabi [94] highlights that patients with incurable diseases go through a process of accepting their illness, moving through stages such as shock, denial, anger, and depression before reaching acceptance. This further explains why this need is especially challenging to meet for palliative care patients. Acceptance of missed opportunities and past life decisions were also identified as inadequately met spiritual needs that nurses found to be the least properly satisfied among palliative patients. According to a qualitative study by Wright et al. [95] that examined the attitudes of 15 terminally ill patients toward dying, palliative patients frequently consider lost chances and past mistakes, and see illness as an opportunity for change, personal development, and a fresh appreciation of life and the present moment.

Interestingly, our study found no significant difference in the way nurses assessed patient needs based on whether they felt adequately trained or not. Both nurses who consider themselves adequately trained in palliative care and those who do not equally assess the importance of palliative patients’ needs and their satisfaction. This is surprising because more training in palliative care is usually associated with greater knowledge [96], but it can suggest that the evaluation of patient needs and satisfaction can be consistent regardless of the nurses’ self-assessed level of palliative care education. The fact that those two groups of nurses assess patient needs similarly suggests that formal education might not have such a strong influence on their clinical judgment, or nurses may share a professional understanding of patient needs. More research is needed to see if practical experience or workplace routines have a bigger impact than their view of their education.

### Strengths and Limitations

The strength of this study lies in the use of questionnaires validated on a population of Croatian nurses, which demonstrates strong psychometric properties. An additional strength lies in the contextual relevance of the instruments, as they were specifically validated within the Croatian nursing population, enhancing their applicability and sensitivity to local healthcare dynamics.

This study has several methodological limitations. Firstly, it relied on a convenience sample of nurses from a single geographic area, which may limit the extent to which the findings can be applied to other regions or healthcare settings. But even though the study focused on a single region, it included participants from different institutions (hospital, hospice, health centre), which adds some diversity to the sample. Also, the sample size of 237 nurses is still a strength in terms of statistical power. Moreover, all participants were female, which introduces the possibility of gender bias in the validation process. Although all participants were female, this likely reflects the gender distribution in the Croatian nursing workforce; it may limit the generalizability of findings to male nurses. The cross-sectional design is another limitation, as it does not allow for tracking changes in nurses’ perceptions over time or with increased palliative care experience. Additionally, self-assessment tools have inherent weaknesses, such as capturing only the current state (which may not be stable), potential insincerity in responses, and the tendency to give socially desirable answers. Beyond these general issues, there are specific challenges associated with using such instruments to assess patients’ needs from the perspective of healthcare professionals. These include potential biases due to limited understanding of patients’ personal experiences, difficulty in fully capturing subjective aspects of patient care, and communication barriers, especially in emotionally sensitive areas like spirituality or psychological distress. These limitations should be carefully considered when designing future studies of a similar nature.

The findings of this study have significant applications. They demonstrate that improving palliative care necessitates targeted interventions, improved education, and strengthened collaboration. To learn more about palliative patients’ needs and how effective palliative care is, future research should also incorporate the opinions of patients, families, and other medical professionals.

## 5. Conclusions

This study highlights significant discrepancies between the perceived psychological and spiritual needs of palliative patients and the extent to which these needs are met. Despite some educational exposure, most nurses feel underprepared, emotionally exhausted, and unsupported, while acknowledging the critical role of interdisciplinary care teams.

The contribution of this study to nursing science lies in a deeper understanding of the multidimensional needs of patients within the specific sociocultural context of Croatia, thereby enriching the theoretical framework of holistic nursing care. In terms of nursing research, the findings provide a foundation for future studies, particularly longitudinal research examining relationships between systemic deficiencies, professional burnout, and patient outcomes. Regarding nursing education, the results highlight gaps in preparation and underscore the need for enhanced educational content addressing psychological, social, and spiritual aspects of palliative care.

Based on these findings, several key recommendations emerge. First, palliative care education should be strengthened at undergraduate, postgraduate, and lifelong learning levels, with greater emphasis on the psychological, social, and spiritual dimensions of care. Second, healthcare institutions should provide emotional and professional support for nurses working in palliative settings to reduce emotional exhaustion and prevent burnout. Finally, greater support and education should be offered to family caregivers, who currently bear much of the responsibility for care, particularly in home-based settings. Implementing these recommendations may help reduce unmet needs identified in this study, improve the quality of palliative care delivery, and promote dignified end-of-life care for patients and their families.

## Figures and Tables

**Table 1:** Nurses’ experiences and perceptions in palliative care.

	Total Sample (*N* = 237)	Nurses’ Perception of Adequate Training		*p* Value ^b^
		Yes (*n* = 91)	No (*n* = 143)	
Years of experience in palliative care ^a^	7 (14)	10 (11.5)	5 (15)	<0.001
Nurses’ education and experience in palliative care; *n* (%)				
Working with palliative patients is a part of regular duties	156 (65.8)	79 (86.8)	76 (53.1)	<0.001
Attended lectures and passed exams in the field of palliative care during formal education	162 (68.4)	69 (75.8)	93 (65.0)	0.081
Participated in lifelong learning programs in the field of palliative care after completing formal education	92 (38.8)	52 (57.1)	40 (27.9)	<0.001
Sometimes in need of support to better respond to the patients’ problems due to emotionally exhausting work with palliative patients	172 (72.6)	78 (85.7)	94 (66.2)	<0.001
Nurses’ perceptions and attitudes about palliative care; *n* (%)				
Due to the emotional demands of their job, professionals in palliative care need various forms of support, including psychological assistance	220 (92.8)	85 (93.4)	135 (94.4)	0.735
Secondary healthcare schools and universities should offer more courses in palliative medicine	201 (84.8)	75 (82.4)	126 (88.1)	0.222
It is essential to organize conferences more regularly to present knowledge in the field of palliative medicine	178 (75.1)	72 (93.5)	106 (95.5)	0.550
Palliative care should be provided by an interdisciplinary team of experts (e.g., doctors, nurses, psychologists, priests, social workers, etc.)	227 (95.8)	88 (96.7)	139 (97.2)	0.827
In our healthcare and social care system, there are palliative care teams that are sufficient for the care of palliative patients	110 (46.4)	53 (58.2)	57 (39.8)	0.006
The responsibility of caring for the needs of palliative patients often falls primarily on their families	214 (90.3)	82 (90.1)	132 (93.6)	0.330

^a^ Median and interquartile Range; ^b^ Mann–Whitney U test for years of experience; Chi-square test for other variables.

**Table 2:** Comparison of nurses’ assessment of palliative patients’ psychological needs and their satisfaction levels.

Palliative Patients’ Psychological Needs	Assessment of Importance Level		Assessment of Satisfaction Level		Wilcoxon Signed Ranks Test	
	Median	IQR ^a^	Median	IQR ^a^	Z Score	*p* Value
Need to overcome depressive mood	4.0	1.0	3.0	2.0	−8.698	<0.001
Need to experience pleasure	4.0	1.0	3.0	1.0	−8.523	<0.001
Need to reduce fear of physical suffering	4.0	2.0	3.0	1.0	−9.368	<0.001
Need to reduce fear of medical treatment	4.0	1.0	3.0	1.0	−8.010	<0.001
Need to reduce fear of disease progression	4.0	2.0	3.0	1.0	−9.219	<0.001
Need to reduce fear of loneliness	4.0	2.0	3.0	1.0	−9.208	<0.001
Need to reduce fear of death	4.0	2.0	3.0	1.0	−9.474	<0.001
Need for alleviating the fear of the unpredictability of the future	4.0	2.0	3.0	1.0	−9.257	<0.001
Need for open expression of emotions	4.0	1.0	3.0	1.0	−7.605	<0.001
Need to stop experiencing guilt	3.0	1.0	3.0	1.0	−5.672	<0.001
Need to avoid feelings of shame	3.0	1.0	3.0	1.0	−5.667	<0.001
Need for emotional control	4.0	1.0	3.0	1.0	−7.743	<0.001
Need for accepting changes in physical appearance	4.0	1.0	3.0	1.0	−8.427	<0.001
Need for the ability to see the positive sides of the situation	4.0	1.0	3.0	1.0	−8.073	<0.001
Need to alleviate feelings of being overwhelmed by all the decisions that need to be made	4.0	1.0	3.0	1.0	−7.756	<0.001
Need to reduce frustration due to difficulty in performing tasks they previously did	4.0	1.0	3.0	1.0	−8.524	<0.001
Need for control over their own body	4.0	2.0	3.0	1.0	−8.497	<0.001
Need for control over their own life	4.0	2.0	3.0	1.0	−8.522	<0.001
Need to seek help	4.0	1.0	3.0	1.0	−7.553	<0.001
Need for more effective decision-making	4.0	1.0	3.0	1.0	−7.061	<0.001

^a^ Interquartile range.

**Table 3:** Comparison of nurses’ social assessment of palliative patients’ social needs and their satisfaction levels.

Palliative Patients’ Social Needs	Assessment of Importance Level		Assessment of Satisfaction Level		Wilcoxon Signed Ranks Test	
	Median	IQR ^a^	Median	IQR ^a^	Z Score	*p* Value
Need for structuring the day	3.0	1.0	3.0	1.0	−7.659	<0.001
Need for relaxation	4.0	1.0	3.0	1.0	−8.370	<0.001
Need for employment or continuing education ^b^	4.0	1.0	3.0	1.0	−9.264	<0.001
Need for the safety of children (who will take care of them and look after them)	4.0	1.0	3.0	1.0	−9.435	<0.001
Need for connection with a partner	3.0	1.0	3.0	1.0	−6.769	<0.001
Need to talk about the illness with a partner	3.0	1.0	3.0	1.0	−5.458	<0.001
Need for a close relationship with children/child	3.0	1.0	3.0	1.0	−5.232	<0.001
Need for a close relationship with family, friends, neighbors, or colleagues	3.0	1.0	3.0	1.0	−4.883	<0.001
Need to avoid talking about the illness because they do not want to burden others	4.0	1.0	3.0	1.0	−6.881	<0.001
Need to talk to others about the illness	3.0	1.0	3.0	1.0	−6.386	<0.001
Need to reconcile different opinions regarding the type of treatment that should be applied	3.0	1.0	3.0	1.0	−6.424	<0.001
Need for more support from others	3.0	1.0	3.0	1.0	−6.262	<0.001
Need to find a trusted person to talk to about the illness	3.0	1.0	3.0	1.0	−5.500	<0.001
Need for more practical help from a partner or family	3.0	1.0	3.0	1.0	−2.776	0.006
Need for others to burden them less with their own worries	3.0	1.0	3.0	1.0	−3.931	<0.001
Need for significant others not to dramatize the situation	3.0	1.0	3.0	1.0	−5.400	<0.001
Need for others not to deny the seriousness of the situation	3.0	1.0	3.0	1.0	−5.168	<0.001
Need to overcome loneliness	4.0	1.0	3.0	1.0	−8.098	<0.001
Need not to be abandoned by others	3.0	1.0	3.0	1.0	−5.770	<0.001
Need for more efficient completion of routine activities	4.0	1.0	3.0	1.0	−7.357	<0.001
Need to continue social activities	4.0	1.0	3.0	1.0	−7.332	<0.001
Need to accept delegating tasks to others due to the inability to continue performing them	3.0	1.0	3.0	1.0	−6.445	<0.001
Need to accept dependence on others	4.0	1.0	3.0	1.0	−8.421	<0.001

^a^ Interquartile range; ^b^ Does not apply to patients with dementia or in the terminal stage.

**Table 4:** Comparison of nurses’ assessment of palliative patients’ spiritual needs and their satisfaction levels.

Palliative Patients’ Spiritual Needs	Assessment of Importance Level		Assessment of Satisfaction Level		Wilcoxon Signed Ranks Test	
	Median	IQR ^a^	Median	IQR ^a^	Z Score	*p* Value
Need to engage in meaningful activities	3.0	1.0	3.0	1.0	−6.965	<0.001
Need to make oneself available to others	4.0	1.0	3.0	1.0	−7.299	<0.001
Need to maintain trust in God or religion	3.0	1.0	3.0	1.0	−5.714	<0.001
Need to find meaning in death	4.0	1.0	3.0	1.0	−7.245	<0.001
Need to accept the illness	4.0	1.0	3.0	1.0	−8.164	<0.001
Need to accept lost time or opportunities in life	4.0	1.0	3.0	1.0	−7.306	<0.001
Need to accept wrong life choices that cannot be corrected	4.0	1.0	3.0	1.0	−7.415	<0.001
Need to accept the impossibility of reconciling with certain people who have passed through their life	3.0	1.0	3.0	1.0	−7.740	<0.001
Need to seek spiritual assistance due to fear of death	3.0	1.0	3.0	1.0	−4.714	<0.001
Need to receive the sacrament of anointing of the sick without associating it with last rites or imminent death	3.0	2.0	3.0	2.0	−1.104	0.270

^a^ Interquartile range.

## Data Availability

The data presented in this study are available on request from the corresponding author due to ethical reasons.

## Supplementary Materials

Questionnaires available upon request. The following supporting information can be downloaded at: https://www.mdpi.com/article/10.3390/nursrep16010029/s1, Table S1: Differences in the assessment of palliative patients’ social needs by nurses who felt adequately trained in palliative care versus those who felt inadequately trained; Table S2: Differences in the assessment of palliative patients’ psychological needs by nurses who felt adequately trained in palliative care versus those who felt inadequately trained; Table S3: Differences in the assessment of palliative patients’ spiritual needs by nurses who felt adequately trained in palliative care versus those who felt inadequately trained.

## Public Involvement Statement

No public involvement in any aspect of this research.

## Guidelines and Standards Statement

This manuscript was drafted against the STROBE (Strengthening the Reporting of Observational Studies in Epidemiology) reporting guidelines for cross-sectional research.
