# Supplementary material for: Nurses’ Perspectives on Unmet Social, Psychological, and Spiritual Needs of Palliative Patients in Croatia: A Cross-Sectional Study
Source: Nurs Rep. 2026 Jan 16;16(1):29. doi: 10.3390/nursrep16010029 (PMC12844772; doi:10.3390/nursrep16010029)
Supplement: Supplementary file 1 [file nursrep-16-00029-s001.zip › nursrep-3997410-supplementary.pdf]

Table S1. Differences in the assessment of palliative patients' social needs by nurses who felt adequately trained in palliative care versus those who felt inadequately trained

Palliative patients' social needs	Felt adequately trained[a]	Mdn[b]	IQR[c]	Assessment of importance level[d]		Mdn[b]	IQR[c]	Assessment of satisfaction level[d]	
				Z score	P value			Z score	P value
Need for structuring the day	No	4.0	1.0	-0.554	0.579	3.0	1.0	-0.600	0.549
	Yes	3.0	1.0			3.0	1.0		
Need for relaxation	No	4.0	1.0	-0.430	0.667	3.0	1.0	-0.137	0.891
	Yes	4.0	1.0			3.0	1.0		
Need for employment or continuing education[e]	No	4.0	1.0	-0.005	0.996	2.0	1.0	-2.251	0.024
	Yes	4.0	1.0			3.0	1.0		
Need for the safety of children (who will take care of them and look after them)	No	3.0	1.0	-1.238	0.216	3.0	1.0	-1.456	0.145
	Yes	3.0	1.0			3.0	3.0		
Need for connection with a partner	No	3.0	1.0	-0.998	0.318	3.0	1.0	-0.518	0.604
	Yes	3.0	1.0			3.0	1.0		
Need to talk about the illness with a partner	No	3.0	1.0	-1.293	0.196	3.0	1.0	-0.576	0.565
	Yes	3.0	1.0			3.0	1.0		
Need for a close relationship with children/child	No	3.0	1.0	-1.731	0.083	3.0	1.0	-1.196	0.232
	Yes	3.0	1.0			3.0	1.0		
Need for a close relationship with family, friends, neighbors, or colleagues	No	4.0	1.0	-0.395	0.693	3.0	1.0	-0.665	0.506
	Yes	3.0	1.8			3.0	1.5		
Need to avoid talking about the illness because they don't want to burden others	No	3.0	1.0	-0.030	0.976	3.0	1.0	-0.686	0.492
	Yes	3.0	1.0			3.0	1.0		
Need to talk to others about the illness	No	3.5	1.0	-0.595	0.552	3.0	1.0	-0.025	0.980
	Yes	3.0	1.0			3.0	1.0		
Need to reconcile different opinions regarding the type of treatment that should be applied	No	3.0	1.0	-0.723	0.470	3.0	1.0	-0.683	0.494
	Yes	3.0	1.0			3.0	1.0		
Need for more support from others	No	4.0	1.0	-0.871	0.384	3.0	1.0	-0.757	0.449

	Yes	3.0	1.0			3.0	1.0		
Need to find a trusted person to talk to about the illness	No	3.0	1.0	-1.724	0.085	3.0	1.0	-1.064	0.287
	Yes	3.0	1.0			3.0	0.5		
Need for more practical help from a partner or family	No	3.0	1.0	-0.572	0.567	3.0	2.0	-0.148	0.882
	Yes	3.0	1.0			3.0	1.0		
Need for others to burden them less with their own worries	No	3.0	1.0	-0.872	0.383	3.0	1.0	-0.064	0.949
	Yes	3.0	1.0			3.0	1.0		
Need for significant others not to dramatize the situation	No	3.0	1.0	-0.636	0.525	3.0	1.0	-1.991	0.046
	Yes	3.0	1.0			3.0	1.0		
Need for others not to deny the seriousness of the situation	No	4.0	2.0	-1.538	0.124	3.0	1.0	-1.545	0.122
	Yes	4.0	1.0			3.0	1.0		
Need to overcome loneliness	No	3.0	1.0	-1.274	0.202	3.0	1.0	-1.143	0.253
	Yes	3.0	1.0			3.0	1.0		
Need not to be abandoned by others	No	4.0	2.0	-1.675	0.094	3.0	1.0	-0.382	0.702
	Yes	4.0	1.0			3.0	1.0		
	Yes	3.0	1.0			3.0	1.5		
Need for more efficient completion of routine activities	No	4.0	1.0	-1.793	0.073	3.0	1.0	-0.894	0.371
	Yes	4.0	1.0			3.0	1.0		
Need to continue social activities	No	4.0	1.0	-0.918	0.359	3.0	1.0	-1.806	0.071
	Yes	4.0	1.0			3.0	1.0		
Need to accept delegating tasks to others due to the inability to continue doing them	No	4.0	1.0	-1.843	0.065	3.0	1.0	-0.304	0.761
	Yes	3.0	1.0			3.0	1.0		
Need to accept dependence on others	No	4.0	2.0	-1.232	0.218	3.0	1.0	-0.281	0.778
	Yes	4.0	1.0			3.0	1.0		
	Yes	4.0	1.0			3.0	1.0		

[a]Yes: n=91, No: n=143
[b]Median
[c]Interquartile range
[d]Mann-Whitney U test
[e]Does not apply to patients with dementia or in the terminal stage

Table S2. Differences in the assessment of palliative patients' psychological needs by nurses who felt adequately trained in palliative care versus those who felt inadequately trained

**Palliative patients' psychological needs**	**Felt adequately trained[a]**	**Mdn[b]**	**IQR[c]**	**Assessment of importance level[d]**		**Mdn[b]**	**IQR[c]**	**Assessment of satisfaction level[d]**	
				**Z score**	**P value**			**Z score**	**P value**
Need to overcome depressive mood	No	4.0	1.0	-0.959	0.338	3.0	2.0	-0.869	0.385
	Yes	4.0	1.0			3.0	1.5		
Need to experience pleasure	No	4.0	2.0	-1.637	0.102	3.0	1.0	-0.858	0.391
	Yes	4.0	2.0			3.0	1.0		
Need to reduce fear of physical suffering	No	4.0	1.0	-2.120	0.034	3.0	1.0	-0.997	0.319
	Yes	3.0	1.0			3.0	1.0		
Need to reduce fear of medical treatment	No	4.0	2.0	-1.419	0.156	3.0	1.0	-1.216	0.224
	Yes	4.0	2.0			3.0	1.0		
Need to reduce fear of disease progression	No	4.0	2.0	-1.151	0.250	3.0	1.0	-1.548	0.122
	Yes	4.0	2.0			3.0	1.0		
Need to reduce fear of loneliness	No	4.0	2.0	-0.836	0.403	3.0	1.0	-1.307	0.191
	Yes	4.0	2.0			3.0	1.0		
Need to reduce fear of death	No	4.0	2.0	-0.837	0.402	3.0	1.0	-1.704	0.088
	Yes	4.0	2.0			3.0	1.0		
Need for alleviating the fear of the unpredictability of the future	No	4.0	1.0	-0.884	0.377	3.0	1.0	-1.695	0.090
	Yes	3.0	1.0			3.0	1.0		
Need for open expression of emotions	No	3.0	1.0	-0.818	0.413	3.0	1.0	-1.074	0.283
	Yes	3.0	1.0			3.0	1.0		
Need to stop experiencing guilt	No	3.0	1.0	-0.107	0.914	3.0	1.0	-0.219	0.826
	Yes	3.0	1.0			3.0	1.0		
Need to avoid feelings of shame	No	4.0	1.0	-0.726	0.468	3.0	1.0	-0.716	0.474
	Yes	3.0	1.0			3.0	1.0		
Need for emotional control	No	4.0	1.0	-0.825	0.409	3.0	1.0	-0.933	0.351

	Yes	4.0	1.0			3.0	1.0		
Need for accepting changes in physical appearance	No	4.0	1.0	-0.736	0.462	3.0	1.0	-1.195	0.232
	Yes	3.0	1.0			3.0	1.0		
Need for the ability to see the positive sides of the situation	No	4.0	1.0	-0.181	0.856	3.0	1.0	-0.672	0.502
	Yes	4.0	1.0			3.0	1.0		
Need to alleviate feelings of being overwhelmed by all the decisions that need to be made	No	3.0	1.0	-0.232	0.817	3.0	1.0	-0.745	0.457
	Yes	3.0	1.0			3.0	1.0		
	Yes	4.0	1.0			3.0	1.0		
Need to reduce frustration due to difficulty in performing tasks they previously did	No	4.0	2.0	-1.575	0.115	3.0	1.0	-0.928	0.354
	Yes	4.0	1.0			3.0	1.0		
Need for control over their own body	No	4.0	2.0	-1.358	0.174	3.0	1.0	-0.852	0.394
	Yes	3.5	2.0			3.0	1.0		
Need for control over their own life	No	4.0	2.0	-1.703	0.089	3.0	1.0	-1.161	0.245
	Yes	4.0	1.0			3.0	1.0		
Need to seek help	No	4.0	1.0	-1.411	0.158	3.0	1.0	-0.235	0.814
	Yes	3.0	1.0			3.0	1.0		
Need for more effective decision-making	No	4.0	1.0	-0.776	0.438	3.0	1.0	-0.833	0.405
	Yes	4.0	1.0			3.0	1.0		

[a]Yes: n=91, No: n=143
[b]Median
[c]Interquartile range
[d]Mann-Whitney U test

Table S3. Differences in the assessment of palliative patients' spiritual needs by nurses who felt adequately trained in palliative care versus those who felt inadequately trained

**Palliative patients' spiritual needs**	**Felt adequately trained[a]**	**Mdn[b]**	**IQR[c]**	**Assessment of importance level[d]**		**Mdn[b]**	**IQR[c]**	**Assessment of satisfaction level[d]**	
				**Z score**	**P value**			**Z score**	**P value**
Need to engage in meaningful activities	No	4.0	1.0	-0.005	0.996	3.0	1.0	-1.362	0.173
	Yes	3.0	1.0			3.0	1.0		

Need to make oneself available to others	No	3.0	1.0	-0.856	0.392	3.0	1.0	-0.977	0.329
	Yes	3.0	1.0			3.0	1.0		
Need to maintain trust in God or religion	No	4.0	1.0	-0.859	0.390	3.0	1.0	-1.045	0.296
	Yes	3.0	1.0			3.0	1.0		
Need to find meaning in death	No	4.0	1.0	-1.260	0.208	3.0	1.0	-0.083	0.934
	Yes	4.0	1.0			3.0	1.0		
Need to accept the illness	No	4.0	1.0	-0.627	0.531	3.0	1.0	-0.639	0.523
	Yes	3.0	1.0			3.0	1.0		
Need to accept lost time or opportunities in life	No	4.0	1.0	-0.562	0.574	3.0	1.0	-0.314	0.753
	Yes	3.5	1.0			3.0	1.0		
Need to accept wrong life choices that cannot be corrected	No	4.0	1.0	-0.834	0.404	3.0	1.0	-1.010	0.312
	Yes	3.0	1.0			3.0	1.0		
Need to accept the impossibility of reconciling with certain people who have passed through their life	No	3.0	1.0	-1.330	0.183	3.0	1.0	-0.249	0.803
	Yes	3.0	1.0			3.0	1.0		
Need to seek spiritual assistance due to fear of death	No	3.0	1.0	-1.810	0.070	3.0	1.0	-0.166	0.868
	Yes	3.0	2.0			3.0	1.0		
Need to receive the sacrament of anointing of the sick without associating it with last rites or imminent death	No	4.0	1.0	-1.953	0.051	3.0	2.0	-0.314	0.754
	Yes	3.0	1.0			3.0	1.5		
	Yes	4.0	1.0			3.0	1.0		

[a]Yes: n=91, No: n=143
[b]Median
[c]Interquartile range
[d]Mann-Whitney U test
